# Crystallography is more than crystal structures

**DOI:** 10.1107/S2052252515015626

**Published:** 2015-08-22

**Authors:** Sine Larsen

**Affiliations:** a Department of Chemistry, University of Copenhagen, Universitetsparken 5, Copenhagen, 2100, Denmark

**Keywords:** crystallography, editorial, neutrons, synchrotron radiation

## Abstract

New developments in neutron and synchrotron science and technology are discussed.

The triennial IUCr congresses have always been highlights and sources of scientific inspiration in my years as a crystallographer, because not only do they provide the latest results in your own field of interest but they also give an overview of developments of crystallography in all its facets. The 23rd IUCr Congress in Montreal was no exception, and it was particularly festive as it was held in the International Year of Crystallography. New research results based on experiments performed with neutrons and synchrotron radiation have always constituted an important component of the Congress. The use of neutrons and synchrotron radiation has increased over the years not only as measured by the number of experiments performed but also by the number of different experimental techniques that are employed. The large research infrastructures are ideal places for crystallographic experiments – not only do they provide neutrons and X-rays but also the technical expertise that enables complex experiments with a variety of techniques.

The papers submitted to **IUCrJ** for the neutron and synchrotron science and technology section reflect the scientific developments observed at IUCr Congresses. A new X-ray technique, rotational X-ray tracking (RXT), has been developed by Liang *et al.* (2014 ▸). They used this technique to study the temperature dependence of a colloidal gel formed by small crystalline alumina particles in decanoic acid, and demonstrated the applicability of RXT for investigation of the viscoelastic properties of Newtonian, and non-Newtonian liquids, solids and their phase transitions.

The number of experiments using advanced grazing-incidence techniques for the analysis of soft-matter materials has grown hugely in recent years, owing to progress in instrumentation and data analysis. The **IUCrJ** feature article by Hexemer & Müller-Buschbaum (2015 ▸), *Advanced grazing-incidence techniques for modern soft-matter analysis*, is therefore timely. In this review the authors describe how advanced grazing-incidence small- and wide-angle X-ray and neutron scattering (GISAXS, GIWAXS, GISANS and GIWANS) can be used to investigate the morphology of soft-matter materials, the overview being illustrated by excellent examples. Method developments have made it possible to characterize complex soft-matter samples in-depth morphologically. The use of focused micro- to nanometer-sized X-ray beams for the GISAXS and GIWAXS experiments has opened up the study of smaller samples as well as samples in a complex sample environment, like GISAXS with a microfluidic cell. Furthermore, a smaller beam enables very local scattering experiments, which makes it feasible to perform structure determinations of inhomogenous samples.

Small-angle X-ray and neutron scattering (SAXS and SANS) methods have also attained great popularity in the crystallographic community for studies of all types of materials. A growing research area is protein solution SAXS, which is a source of structural information for proteins that are difficult to crystallize. In another feature article, *Beyond simple small-angle X-ray scattering: developments in online techniques and sample environments* by Bras, Koizumi & Terrill (2014 ▸), an overview is given of the development of experimental facilities for SAXS experiments at synchrotron radiation facilities. Dedicated SAXS/WAXS (wide-angle X-ray scattering) beamlines are present at most synchrotron radiation facilities and the use of the complementary information available from simultaneous measurement of SAXS and WAXS in time-resolved studies is described well. SAXS and WAXS measurements become even more powerful if they are supported by other types of measurements, and a thorough description of technique combinations and sample environments is also given in the paper by Bras *et al.* (2014 ▸).

The advances in SAXS are also shown in other sections of **IUCrJ**, as illustrated by the following examples. In the materials and computation section, the paper *Advanced ensemble modelling of flexible macromolecules using X-ray solution scattering* by Tria *et al.* (2015 ▸) describes new developments in the methods for studying unstructured and flexible molecules by SAXS. The biology and medicine section of **IUCrJ** contains examples of the use of SAXS in complex biological systems (Tian *et al.*, 2015 ▸).

Synchrotron radiation has contributed immensely to the development of structural biology. Therefore, it is worth mentioning the paper by Helliwell & Mitchell (2015 ▸) in the biology and medicine section. The authors provide both an excellent overview of the present state of instrumentation, automation and data analysis and an outlook on future developments.

The experiments performed with free electron lasers (FELs) on small crystals of proteins have materialized in a new field ‘serial crystallography’ (Chapman *et al.*, 2011 ▸). A very informative review covering the first five years of this field has been written by Ilme Schlichting (2015 ▸). Advanced instrumentation has been used for the measurement of serial crystallographic data, but handling of the sparse data obtained from the experiment seems to be equally important; Ayyer *et al.* (2015 ▸) show how use of the appropriate algorithm makes it possible to generate crystallographic intensities from sparse data, a result that may have a great impact on the development of synchrotron radiation serial crystallography.

*X-ray techniques for innovation in industry* is the title of a feature article in the neutron and synchrotron section authored by Lawniczak-Jablonska & Cutler (2014 ▸). This paper is a source of inspiration for increased collaborations/interactions between companies and researchers at universities and research institutions. The experimental set-up at present synchrotron facilities provides unique opportunities for the characterization of all types of materials by a variety of techniques: spectroscopy, diffraction, imaging and scattering. In the paper the authors describe how the needs of industry can be met by synchrotron facilities and how this has been stimulated by a project supported by the European Union. The paper illustrates the present level of industrial collaborations and the great potential for enhancing them in the future.

The large neutron and X-ray facilities are serving a wide range of scientific communities extremely well. Synchrotron radiation has had a tremendous impact on the characterization of all types of materials from hard to biological and has contributed to the development of new materials. Scientists in a broad range of fields are familiar with the use of photons generated by third generation storage rings. However, there are also some experiments that could benefit if the beam of photons had a higher brightness and smaller emittance, and other experiments that depend strongly on the coherence properties of the beam. These wishes of the scientists are likely to be fulfilled when the next generation of light sources becomes operational in the near future. In the paper *The potential of future light sources to explore the structure and function of matter* by Edgar Weckert (2015 ▸), the author provides the technical background for the performance of the new light sources that are under construction in Sweden (MAX IV) and Brazil (SIRIUS) in a comparison with the performance of free electron laser facilities. The paper also gives a good overview of the scientific opportunities in terms of new types of experiments that can benefit from the higher brightness, the smaller emittance and the coherence of the beam. The first of the new generation of light sources, MAX IV, starts operating in 2016 and one could expect that this will lead to new technical developments and new scientific results for publication in **IUCrJ**.
